# Hybrid Mineral/Organic Material Induces Bone Bridging and Bone Volume Augmentation in Rat Calvarial Critical Size Defects

**DOI:** 10.3390/cells11182865

**Published:** 2022-09-14

**Authors:** Marie Dubus, Loïc Scomazzon, Charlotte Ledouble, Julien Braux, Abdelilah Beljebbar, Laurence Van Gulick, Adrien Baldit, Caroline Gorin, Halima Alem, Nicole Bouland, Marissa Britton, Jessica Schiavi, Ted J. Vaughan, Cédric Mauprivez, Halima Kerdjoudj

**Affiliations:** 1 Biomatériaux et Inflammation en Site Osseux (BIOS), Université de Reims Champagne Ardenne, EA 4691 Reims, France; 2 UFR d’Odontologie, Université de Reims Champagne Ardenne, 51100 Reims, France; 3 Pôle Médecine Bucco-Dentaire, Hôpital Maison Blanche, Centre Hospitalier Universitaire de Reims, 51100 Reims, France; 4 BioSpecT EA 7506, Université de Reims Champagne Ardenne, 51100 Reims, France; 5 UFR de Pharmacie, Université de Reims Champagne Ardenne, 51100 Reims, France; 6 Ecole Nationale d’Ingénieurs de Metz, CNRS, LEM3, Université de Lorraine, 57078 Metz, France; 7 URP2496, Pathologies, UFR Odontologie, Imagerie et Biothérapies Orofaciales et Plateforme Imagerie du Vivant, Université Paris Cité, 92120 Montrouge, France; 8 AP-HP, Services Médecines Bucco-Dentaire (GH Paris Sud-Sorbonne Université), 92120 Montrouge, France; 9 CNRS, IJL, Université de Lorraine, 54500 Nancy, France; 10 Service d’Anatomo-Pathologie, Université de Reims Champagne Ardenne, 51100 Reims, France; 11 Biomechanics Research Centre (BioMEC), Biomedical Engineering, School of Engineering, College of Science and Engineering, National University of Ireland, H91 HX31 Galway, Ireland

**Keywords:** dicalcium phosphate dihydrate, polysaccharides, hybrid bioactive materials, critical-sized bone defect, bone volume augmentation, multi-scale characterization

## Abstract

In craniofacial bone defects, the promotion of bone volume augmentation remains a challenge. Finding strategies for bone regeneration such as combining resorbable minerals with organic polymers would contribute to solving the bone volume roadblock. Here, dicalcium phosphate dihydrate, chitosan and hyaluronic acid were used to functionalize a bone-side collagen membrane. Despite an increase in the release of inflammatory mediators by human circulating monocytes, the in vivo implantation of the functionalized membrane allowed the repair of a critical-sized defect in a calvaria rat model with de novo bone exhibiting physiological matrix composition and structural organization. Microtomography, histological and Raman analysis combined with nanoindentation testing revealed an increase in bone volume in the presence of the functionalized membrane and the formation of woven bone after eight weeks of implantation; these data showed the potential of dicalcium phosphate dihydrate, chitosan and hyaluronic acid to induce an efficient repair of critical-sized bone defects and establish the importance of thorough multi-scale characterization in assessing biomaterial outcomes in animal models.

## 1. Introduction

In clinical practice, craniofacial bone regeneration is challenging due to the lower regeneration rate of flat bones in comparison with long bones. Severe bone defects in the jaw, caused by tooth extraction or loss, often result in a reduction of the original ridge dimension, hampering the stabilization and the long-term integration of dental implants. Biomaterial-based approaches and guided bone regeneration (GBR) are currently employed for the repair of substantial bone defects as it has been shown to be very effective for horizontal and vertical defect augmentations [1]. Therefore, the application of a physical biodegradable barrier in the GBR procedure plays a key role in preventing the ingrowth of cells from the surrounding epithelium and connective tissue and favoring osteoprogenitor cells to proliferate and form new bone tissue. During the last decades, different biomaterials of biological or synthetic origin have been designed, aiming to favor new bone formation. Calcium phosphate (CaP) materials have given rise to a lucrative market in regenerative materials because they encourage bone growth. CaPs are made from the combination of PO_4_ anions, with Ca cations in different “Ca:P” ratios, that are, 1.67 for hydroxyapatite (HA), 1.5 for α-and β-tricalcium phosphate (TCP) and 1.0 for dicalcium phosphate dihydrate (DCPD) and dicalcium phosphate anhydrate (DCPA) [2]. The solubility of CaPs-based material in physiological solution is of great importance because it can predict the in vivo resorption and osteoprogenitor cell stimulation. While biocompatible HA/β-TCP mixture exhibits a very low solubility at physiological conditions and an extremely slow in vivo degradation [3], DCPD showed a sustained in vivo resorption and de novo bone formation [4,5], increasing, therefore, the interest of researchers in the recent years. We recently described that a DCPD coating induces the commitment of mesenchymal stem cells in vitro into a bone-forming cell lineage without adding osteogenic growth factors [6]. To enhance the regenerative capacity of CaPs and augment cranial repair, many attempts proposed the addition of stem cells, osteogenic growth factors, or cytokines, alone or in a combination (i.e., tissue engineering strategies) [7,8]. Although the field of tissue engineering might have a bright future in regenerative medicine, significant drawbacks such as the lack of reliable efficacy, high costs, potential side effects of the bioactive molecules, possible safety risks, and ethical issues hinder its current implementation in clinical cases. Another route of improvement lies in the optimization of intrinsic features of CaPs-based materials (i.e., structure, roughness, mechanical properties, polymer combination) to enhance the biological response [9]. Hybrid mineral/organic materials are especially beneficial for biomedical applications due to the combined properties of both the organic moiety, such as functionality and flexibility, and the heat resistance and stability of the inorganic component. Bioinspired precipitation of the inorganic part with polymeric additives from an aqueous solution in ambient conditions enables the synthesis of intriguing hybrid CaPs materials that are often similar to biological structures. Besides proteins, protein mimics, and synthetic polymers, polysaccharides have emerged as unique sources for preparing hybrid materials with special properties such as biocompatibility, biodegradability, low density, low thermal conductivity and bioactivity [10,11]. Chemically, polysaccharides may be composed of single monosaccharides or two or more different monomeric units and can acquire positive or negative charges in an aqueous solution. The resulting charged groups are useful for building the networks, controlling the nucleation and increasing the bioactivity of the resulting hybrid materials [12,13]. Chitosan (CHI) and hyaluronic acid (HA) are largely investigated in bone regenerative medicine as CHI forms polycation-polyanion complexes in the presence of HA and its amino groups make it specifically relevant for the biomineralization process. We recently indicated that it is possible to endow CaPs with osteoinductive properties by optimizing the material characteristics and by adding CHI and HA during the nucleation [14,15,16]. The objective of this study was to functionalize the bone-side of the collagenic membrane, currently used in the GBR procedure, with CaP, CHI and HA components. We explored the bone regeneration capacity of the functionalized membrane in the rat calvarial 5-mm critical-size defects with a thorough multi-scale characterization in assessing the membrane outcomes after eight weeks of implantation.

## 2. Material and Methods

### Preparation of the Functionalized Membrane

Sodium dihydrogen phosphate hydrate (NaH_2_PO_4_), calcium chloride hydrate (CaCl_2,_ 2H_2_0), sodium chloride and chitosan (75–85% deacetylated, low molecular weight) from Sigma Aldrich, Saint-Quentin-Fallavier, France and hyaluronic acid (200 kDa) from Lifecore Biomedical, Cheska, MA, USA were used without further purification. Salt solutions were prepared in ultrapure water (Millipore^®^, Burlington, MA, USA). CaCl_2_, 2H_2_O (0.32 M) and chitosan (CHI) 0.3 mg/mL were dissolved in NaCl (0.15 M)/HCl (2 mM) buffer at pH 4 whereas NaH_2_PO_4_ (0.19 M) and hyaluronic acid (HA) 0.3 mg/mL were prepared in NaCl (0.15 M) buffer at pH 10. The build-up was then performed on the Bio-Gide^®^ collagen membrane, (Geistlich Pharma^®^, Wolhusen, Switzerland) as previously described [17]. The biocompatible glue (Bostik^®^, Colombes, France) was used to maintain the functionalized membranes on glass coverslips for in vitro experiments. Raw Bio-Gide^®^ collagen membrane was used as a control for the biological assessments.

## 3. Morphological and Physicochemical Characterizations

### 3.1. Scanning Electron Microscopy with a Field Emission Gun (FEG-SEM)

FEG-SEM investigations were performed with a FEG-SEM (JEOL JSM-7900F, Croissy-sur-Seine, France), on the slide of functionalized membrane sputtered with a thin gold–palladium film (JEOL ion sputter JFC 1100). The functionalized membranes were previously cut neatly with a scalpel blade and placed on the slide for gold-palladium coating. The images were acquired from the secondary electrons at a primary beam energy of 2 kV.

### 3.2. Confocal Raman Spectroscopy Mapping

Raman maps were recorded with a near infrared confocal Raman spectrometer (Labram ARAMIS, Horiba Jobin Yvon S.A.S., Longjumeau, France) coupled to a microscope (Olympus, BX41, Beauvais, France). The microscope was equipped with a xy-motorized (Marzhauser, Wetzlar, Germany), computer-controlled sample stage, which enabled automatic scanning of the sample with a resolution of 1 µm. The excitation source (785 nm) was provided by a diode laser (Toptica Photonics, Gräfelfing, Germany). The laser power on the sample was about 60 mW; this laser light was focused on the coating with a 100 X optimized objective (Olympus, Beauvais, France); this objective collected the scattered light by the sample, which was then analyzed by the spectrometer equipped with a Peltier-cooled charge-coupled device detector. All spectra were acquired using a 20 s integration time in the 700–1800 cm^−1^ spectral region with a spectral resolution of 4 cm^−1^.

### 3.3. Induced Coupled Plasma-Optical Emission Spectroscopy (ICP-OES)

The functionalized membranes were soaked in 1 mL of serum-free Dulbecco’s modified Eagle’s medium (DMEM, Gibco, Darmstadt, Germany) at 37 °C. After 15 min, 2 h, 24 h, and 48 h of incubation, the membranes were retrieved, and the DMEM was collected to assess the calcium and the phosphorus ions using ICP-OES (iCAP 6300 duo plasma emission spectrometer). The results were normalized to serum-free Dulbecco medium.

### 3.4. Transmission Electron Microscopy (TEM)

TEM and High-Resolution TEM (HR-TEM) investigations were performed with a JEOL ARM 200F–Cold FEG TEM/STEM (point resolution 0.19 nm in TEM mode and 0.078 nm in STEM mode) fitted with a GIF Quatum ER. The functionalized membranes were previously soaked in 1 mL of serum-free DMEM at 37 °C for a week, then dehydrated in graded ethanol solutions from 50% to 100% for 10 min and embedded in epoxy resin (48.2% epon 812, 34% anhydride nadic methyl, 16.4% anhydride [2-dodecenyl] succinic, and 1.5% 2, 4, 6-tris dimethy-laminoethyl phenol) for 72 h at 60 °C. Ultra-thin cross-sections (60 nm in thickness) were obtained using an automatic ultramicrotome (Ultracut-UCT Ultramicrotome, Leica, Wetzlar, Germany) at room temperature. The calculation of the d-spacing was performed via the fast Fourier transform.

## 4. Biological Assessments

### Acute Inflammatory Response

Fresh human venous blood was collected in BD Vacutainer^®^ K2E EDTA tubes from healthy donors. Monocytes were isolated from whole blood by using a density gradient centrifugation medium (*v/v*, Granulosep, Eurobio-Abcys, San Diego, CA, USA), then purified by a positive selection with CD14 immunomagnetic beads (MACS, Miltenyi Biotec, Paris, France), according to the manufacturer’s instructions. Resulting CD14 positive monocytes were cultured in RPMI 1640-Glutamax^®^ supplemented with 10% heat-inactivated fetal bovine serum (FBS) and 1% Penicillin/Streptomycin. CD14 positive monocytes were seeded at 5 × 10^5^ cells/mL on UV-decontaminated (20 min) functionalized membranes in a 24 well culture plate and incubated at 37 °C and 5% CO_2_ for 24 h. Lipopolysaccharide (LPS) at 10 ng/mL was used as inflammatory stimulus control. Supernatants were collected and the release of Tumor Necrosis Factor-alpha (TNF-α), Interleukine-1 beta (IL-1β), and Interleukine-10 (IL-10) were measured using human Duoset^®^ (Minneapolis, MN, USA) TNF-α, IL-1β and IL10 (R&D systems, Minneapolis, MN, USA), according to manufacturer’s instructions. Absorbances were measured at 450 nm.

After 24 h of culture, samples were fixed with 2.5% (*w/v*) glutaraldehyde (Sigma-Aldrich) at room temperature for 1 h. Samples were then rinsed twice with PBS, dehydrated in graded ethanol solutions and desiccated in hexamethyldisilazane (Sigma-Aldrich) for 10 min. After air-drying at room temperature, samples were sputtered with a thin gold-palladium and observed with a FEG-SEM (JEOL JSM-7900F, Croissy-sur-Seine, France), and images were acquired from the secondary electrons at a primary beam energy of 5 kV.

## 5. In Vivo and Ex Vivo Assessment

### 5.1. Parietal Bone Implantation

**Figure 1:**
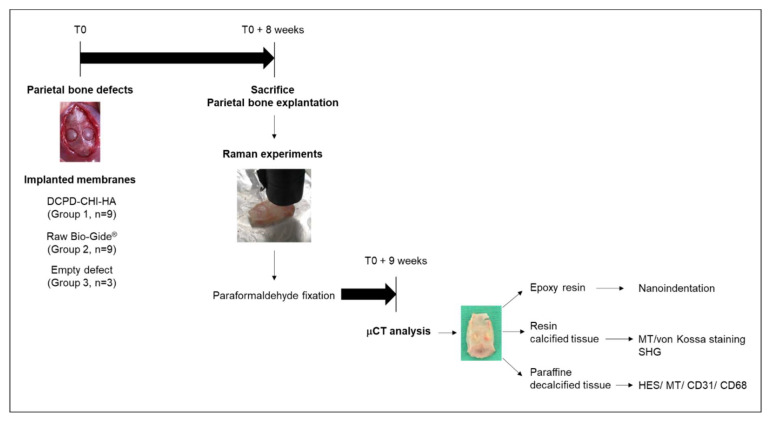
Workflow of in vivo and ex vivo experiments.

In vivo animal tests were carried out following the guidelines approved by the Committee on Animal Care of Reims University (N°2018111612178592). Animals were anesthetized through inhalation of 2.5% isoflurane (Isoflu-Vet^®^ (Saint-Quentin-Fallavier, France) 1000 mg/g, Dechra (Northwich, UK)) and euthanized at the end of each procedure by cervical dislocation after anesthesia with 5% of isoflurane. The procedure was performed on 8-week-old Fisher rats male (*n* = 21). Briefly, after cutaneous disinfection, a linear sagittal incision was made along the top of the skull and two calvaria bone defects were created on each side of the parietal bone with a 5 mm diameter trephine drill at 1500 rpm, under saline irrigation. Right and left bone defects were respectively covered with 5 mm UV-decontaminated functionalized membranes which were extended over the surrounding bone margins. Then, the periosteum and skin were carefully sutured. Animals implanted with the raw Bio-Gide^®^ membranes were used as controls for the in vivo experiments. The bone repair was assessed at 8 weeks postoperatively by Raman analysis, computed microtomography, indentation testing, second harmonic generation and histology (Figure 1).

### 5.2. Raman

Raman spectroscopy analyzes were performed on fresh and hydrated explanted parietal bone. Raman spectra were recorded with HE-785 commercial Raman spectrometer (Jobin-Vyon-Horiba, Longjumeau, France) as previously described [18]. Briefly, this setup consisted in high efficiency (HE) spectrograph with a fixed 950 gr/mm grating coupled to a matrix charge-coupled device (CCD) detector (Andor Technologies, South Windsor, CT, USA); this setup was coupled to a commercially available In Photonics (Inc., Doweny St., CA, USA) fiber optic probe; this fiber probe is composed of a 100-μm excitation fiber and a 200-μm collection fiber. The laser light was transmitted through a 100-μm fiber to the sample; the 200-μm fiber probe collected the scattered signals from the sample and directed them to the spectrometer. The excitation source (785 nm) was provided by OEM diode laser (Process Instruments Inc., Salt Lake City, UT, USA). The output power at the distal end of the excitation fiber was 120 mW. Five to ten spectra were recorded from each sample with 10 sec integration time in the range from 500 to 3200 cm^−1^. Data acquisition was performed using the Labspec 5.0 software (HORIBA Jobin Yvon, Edison, NJ, USA). Data pretreatment consisted of fluorescence background subtraction, cosmic ray removal, wavenumber calibration, instrument response correction, and subsequent linearization of the wavenumber axis. Wavenumber calibration was performed using two Raman calibration standards, 4-acetamidophenol and cyclohexane, along with the emission lines of the neon lamp. The wavelength-dependent signal detection efficiency of the setup was measured using a calibration standard (standard reference material number-2241; NIST, Gaithersburg, MD, USA). Cosmic rays were removed from the spectra. The broad background spectrum due to tissue autofluorescence was subtracted using a fifth-order polynomial fit. Data were smoothed using a seven-point Savitzky-Golay algorithm. The resulting spectra were then normalized using a Standard Normal Variate (SNV) procedure.

### 5.3. Micro Tomography

Explanted parietal bones were fixed in 4% (*w/v* in PBS) paraformaldehyde (Sigma-Aldrich) for one week and imaged using micro-computed tomography scans (Skyscan 1076, Bruker (Billerica, MA, USA)) with the following settings: tube voltage, 80 kV; tube current, 0.135 mA; voxel size 17.9 μm^3^ and 0.5 mm Al filter. Three-dimensional (3D) images were rebuilt, reoriented and analyzed using respectively the NRecon GPU version, Dataviewer and CTAn 1.18 (Bruker) software programs. After 3D reconstruction, bone volumes were segmented using a global threshold obtained by using the mean value of densities leading to segment bone, and only bone, outside of the treated region, retrieved in each scan after manual segmentation. Bone volumes (BV) were measured in a region of interest consisting of a cylinder of 5 mm diameter and 2.5 mm height and manually centered into the defect.

### 5.4. Histology and Immunohistochemistry

The paraformaldehyde-fixed explanted parietal bone provided by four rats was dehydrated in ethanol baths, embedded in resin (Technovit^®^ 9100 (Wehrheim, Germany)), sliced at a 6 µm thickness and stained with Masson’s trichrome (MT) and Von Kossa (Sigma Aldrich).

For the paraffin inclusion, the right parietal bone defects, from four rats, were decalcified for 10 weeks in baths (changed twice a week) of 20 mL of (paraformaldehyde (0.2%), EDTA (4.13%) at pH = 7.4) solution. Dehydrated samples embedded in paraffin were sliced at a 3 µm thickness and stained with Hematoxylin-eosin-saffron (HES) and MT. For the immunohistochemistry, after deparaffinization, sections were incubated with EDTA (pH = 8.4) for 20 min at 97 °C (for CD31 labelling) and with citrate buffer (pH = 6, Dako) for 40 min at 95 °C (for CD68 labelling), before incubation overnight with the primary antibodies (Abcam (Cambridge, UK), dilution 1:1000). The counterstain and post-counterstain comprised hematoxylin. Images were taken using VS 120 OLYMPUS scanner (Shinjuku-ku, Tokyo, Japan).

### 5.5. Confocal Microscopy and Second Harmonic Generation (SHG)

Two-photon excitation laser scanning confocal microscopy and SHG were performed under circular polarization on the bloc of the resin-embedded parietal bone. Images were obtained with a confocal microscope (LSM 710-NLO, Carl Zeiss SAS, Marly-le-Roi, Germany) coupled with a CHAMELEON femtosecond Titanium-Sapphire Laser (Coherent, Santa Clara, CA, USA). Samples were excited at 860 nm and SHG signal was collected in a 420–440 nm spectral window with 20X objective (ON: 0.8). Images were analyzed with Image J.

### 5.6. Nanoindentation

The central parts of the left parietal bone defect, from four rats, were dehydrated in a series of ascending ethanol baths before being embedded vertically in epoxy resin (EpoThin2TM, Beuhler, IL, USA) and placed under a vacuum to allow the epoxy to fill all spaces. Using a low-speed saw (ISOMETTM Low-Speed Saw, Beuhler, IL, USA) and a diamond blade, the test surface, the width of the bone, was exposed and then polished using series of descending diamond suspension pastes (9 µm, 3 µm, 1.5 µm and 0.05 µm) with polishing cloths on a polishing machine (MetaServ^®^ 250 Grinder-Polisher with Vector^®^ LC Power Head, Beuhler, IL, USA). The samples were washed in deionized water using an ultrasonic bath to avoid cross-contamination between the suspension pastes. The nanoindentation was carried out on a NanoIndenter G200 (Keysight Technologies, CA, USA) with load and displacement resolutions of 50 nN and <0.01 nm, respectively. A Berkovich diamond indenter tip with an elastic modulus of 1141 GPa and a Poisson’s ratio of 0.07 was used. The calibration of the machine was performed using fused silica. 10 indents were carried out on each sample, with indents being at least 10 µm away from the edge of the sample and 15 µm from neighboring. The loading profile included two conditioning steps that reached ¼ max load and ½ max load, followed by a third step that reached a maximum load of 20 mN. Multiple loading cycles, as well as a long hold of 120 sec at each of the peak loads, reduce the impacts of time-dependent plasticity [19]. The indenter was held at 10% of the maximum load for 120 sec was used to determine the rate of thermal expansion. Before further data analysis, the thermal drift correction factor was applied to the test displacement data. The data obtained from the indentation tests were analyzed to determine the modulus and hardness of the samples using the in-built Oliver and Pharr method (Keysight NanoSuite 6.2 Software) [20].

### 5.7. Statistical Analysis

All statistical analyzes were performed using GraphPad Prism^®^ software (San Diego, CA, USA). ICP-OES experiment was performed with three independent functionalized membranes. For inflammatory response, monocytes from six independent, healthy blood donors were tested in technical duplicate. In vivo experiments were performed on 21 rats. Raman and Micro-CT were performed on all rats and nanoindentation experiments on four explanted parietal bones. Statistical analyzes were performed using the *t*-test and Mann–Whitney test. For each test, a value of *p* < 0.05 was accepted as a statistically significant *p* (rejection level of the null hypothesis of equal means).

## 6. Result and Discussion

**Figure 2:**
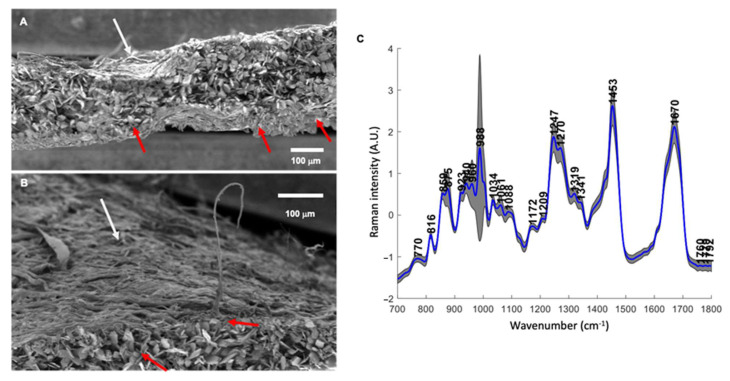
Morphological and physicochemical characterization of coated membrane. (**A**,**B**): Scanning electron microscopy views of coating on full thickness and soft-side, respectively. White and red arrows indicate respectively the collagen fibers and rod-*like* mineral structures, respectively. (Scale bars = 100 μm). (**C**): Raman spectra, revealing the presence of the dicalcium phosphate dehydrate (DCPD) phase.

By using simultaneous spray coating of interacting species process, bone-side collagenic membrane (Bio-Gide^®^; an FDA-approved and commonly applied as a collagen membrane in a broad range of clinical settings) was sprayed with calcium saturated solution supplemented with chitosan (CHI) and phosphate saturated solution supplemented with hyaluronic acid (HA), in the same conditions as previously published (i.e., 50 simultaneous spray cycles for 2 s) [17]. The choice of CHI (polycation) and HA (polyanion) as polyelectrolyte complexes was motivated by their intrinsic antibacterial properties against Gram-positive and Gram-negative bacteria strains [16]. Chitosan/hyaluronic coating showing an unsuccessful build-up and instability in physiological media was rejected as a control. The resulting functionalized membrane was firstly characterized by scanning electron microscopy (SEM). Images showed the presence of mineral structures on the bone-side of the membrane surface but also within the thickness of the membrane (Figure 2A). Rod-*like* shaped mineral occupied the space within the collagen, while the soft tissue side of the Bio-Gide^®^ membrane did not reveal the presence of mineral structures (Figure 2B white arrow). Taken together, these observations suggest the diffusion and the complexation of sprayed species between the collagenous fibers. The chemical composition of the resulting complex was further analyzed by Confocal Raman spectroscopy mapping (Figure 2C). The Raman bands of interest and their corresponding molecular groups were labeled according to literature data [4,21,22]. Spectra highlighted the presence of a strong band at 988 cm^−1^ and 1061 cm^−1^, the signature of the dicalcium phosphate dehydrate (DCPD) phase. Other bands in the spectra were related to the collagen matrix: the amide I vibration at 1670 cm^−1^ was dominated by peptide carbonyl stretching vibration with some contribution of C–N stretching and N–H in-plane bending. The amide III bands at 1270 cm^−1^ and 1247 cm^−1^ were assigned to N–H bending and C–N stretching. The Raman bands at 859 cm^−1^ and 875 cm^−1^ were attributed to proline and hydroxyproline ring vibrations, respectively. The band at 1034 cm^−1^ originated from ν(CC) of proline. The intensity of the specific CHI and HA bands located respectively at 960 cm^−1^ and 1070 cm^−1^ was very weak and superimposed with those of collagen. In addition, the map showing the spatial distribution of the mineral-to-organic ratio (I_988_/I_1670_) was generated and used to assess the interaction between minerals and collagen components (Figure S1—SI); this map revealed that there is considerable heterogeneity in the distribution of mineral products on the collagen membrane.

**Figure 3:**
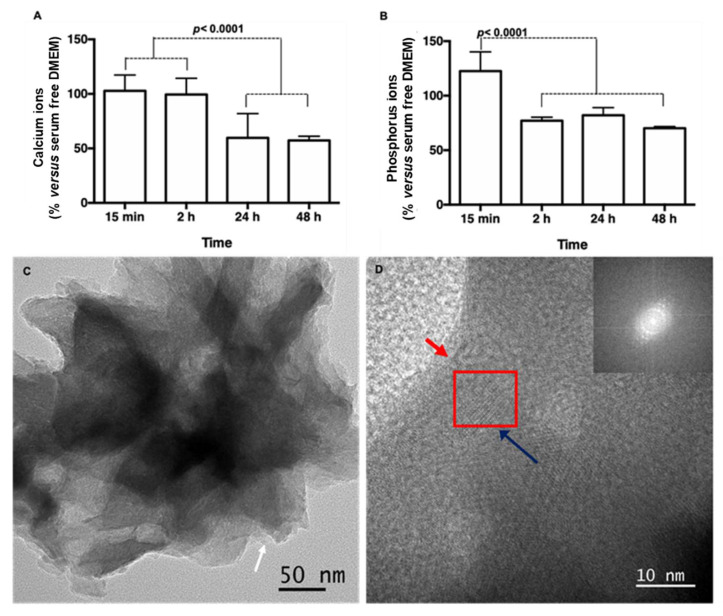
Bioactivity of the functionalized membrane after incubation in the serum-free culture medium. (**A**,**B**): Histograms of the percentage content of, respectively, calcium and phosphorus ions remained in the culture medium analyzed by ICP-OES, showing a significant decrease in Ca and P content 24 h in comparison with 15 min (*n* = 3, *t*-test). (**C**): Transmission electron microscopy and (**D**): High Resolution-Transmission electron microscopy images of the crystal-*like* structure. White, red and blue arrows indicate collagen, chitosan/hyaluronic acid amorphous film and DCPD phase, respectively. The red square shows the hydroxyapatite phase (inserts: electron diffraction, scale bars = 50 and 10 nm).

DCPD has captured the attention of bone reconstructive and regenerative medicine fields in recent years [4,5]. In addition to being one of the precursors of biological apatite present in bone and tooth, the main advantage of DCPD material is its biodegradability which may take place via the dissolution in the physiological liquid and the resorption by osteoclasts. Many studies reported that brushite-based cements could dissolve, resulting in an increase in calcium and phosphate ions in the cell culture medium [5,23]. By using Inductively Coupled Plasma-Optical Emission Spectroscopy (ICP-OES, Waltham, MA, USA), we analyzed the kinetic ions content after 15 min, 2 h, 24 h and 48 h of incubation in serum-free Dulbecco medium (DMEM) [24]. The presented results were normalized to serum-free DMEM in the absence of minerals (Figure 3). A slight increase in phosphorus ions (~120% versus DMEM) was observed after 15 min of soaking, while no increase in calcium ions was noticed. After 24 h a significant decrease in both ions content (above 60%, *p* < 0.0001) was observed (Figure 3A,B). Taken together, we can conclude that the absence of a significant increase in ions is indicative of stable DCPD in the culture media. The decrease in ion content in the culture media could be attributed to the precipitation from the solution, which could lead to the formation of a crystalline phase [24]. To elucidate this hypothesis, High Resolution-Transmission Electron Microscopy (HR-TEM) combined with electron diffraction was also performed on the DMEM incubated membranes. Results indicated the presence of both amorphous but also structures displaying crystalline domain (as highlighted in the red square; Figure 3C,D). The calculation of the d-spacing via the fast Fourier transform indicated the presence of spaces of 0.280, 0.118 and 0.145 nm related to the ($11\bar{2}$), (241), ($42\bar{3}$) plan of the DCPD [25] but also spaces of 0.276, 0.167 and 0.139, corresponding to the (200), (152), (281) plan of the hydroxyapatite [26]. To sum up, bioactive functionalized membranes incubated in physiological condition could convert the DCDP into hydroxyapatite.

**Figure 4:**
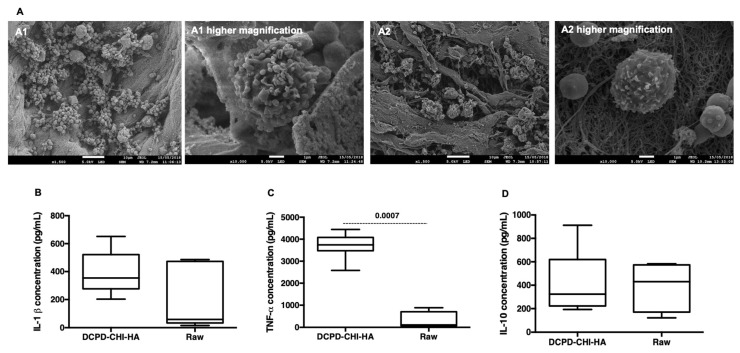
Acute inflammatory response. (**A**): FEG-SEM views of CD14^+^ monocytes in contact with the functionalized (**A1**) and the raw membrane (**A2**) (Scale bars = 100 μm and 10 μm), highlighting the absence of monocyte activation. (**B**–**D**): ELISA analysis of the produced cytokines by CD14^+^ monocytes in the presence of DCPD, CHI and HA components, revealing the pro-inflammatory potential of the material (*n* = 6, Mann–Whitney test).

Currently, the impact of bone materials on the immune response has attracted the attention of many scholars [27,28]. Indeed, a key feature of the healing process is the generation of an optimal environment with moderate inflammatory signals. An exacerbated and/or chronic inflammation may delay and hamper tissue repair. Herein, we sought to investigate the acute response of monocytes in the presence of the functionalized membrane. After 24 h of culture of CD14 positive monocytes, FEG-SEM imaging showed adhered macrophages on the mineral crystals without an apparent activation in comparison with LPS-stimulated monocytes (Figure 4A, Figure S2—SI). The analysis of the released cytokines in the culture supernatant indicated the presence of TNF-α and IL-1β pro-inflammatory cytokines but also the presence of IL-10, an anti-inflammatory cytokine, in all conditions. In comparison with the controls (in the presence and the absence (unpublished data) of the raw membrane), the DCPD-CHI-HA appeared to be pro-inflammatory as a significant increase in TNF-α production was detected (Figure 4B–D). Herein, we thought that the inflammatory action of the functionalized membrane could be attributed to the DCPD phase and rod-shape structure [29] but also to the presence of chitosan [30], as Bio-Gide^®^ membrane is described as inert material [31].

**Figure 5:**
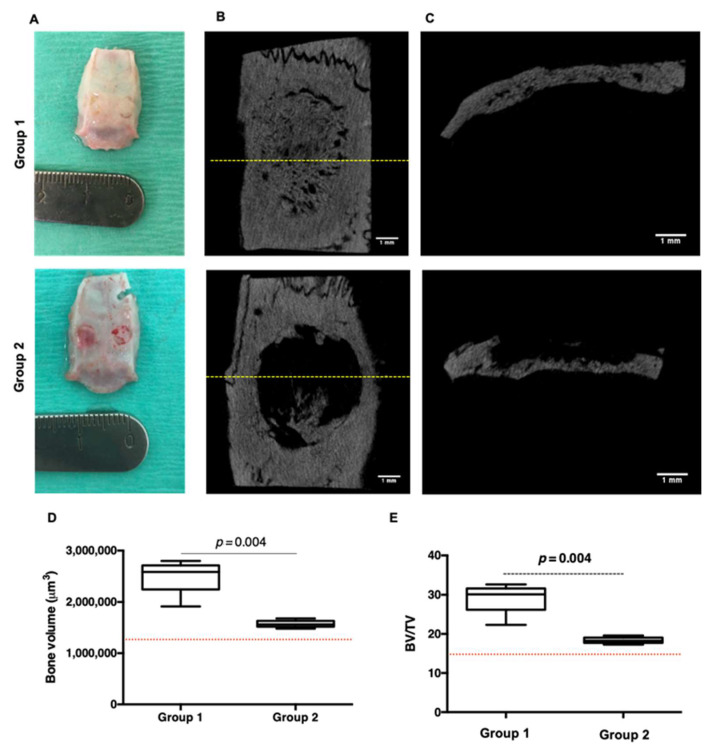
Ex vivo analysis of 8-weeks de novo bone. (**A**): Macroscopical examination of the parietal bone recovery following implantation of the DCPD-CHI-HA (group 1, upper line) and the raw membrane (group 2, lower line). (**B**,**C**): µ-CT coronal and transaxial views of the explanted parietal bone that received the DCPD-CHI-HA (group 1, upper line) and the raw membrane (group 2, lower line) (17.9 μm in resolution). Dashed yellow lines in the coronal views correspond to the transaxial level (Scale bars = 1 mm). (**D**,**E**): Quantitative μ–CT results, indicating respectively the bone volume (BV) and bone volume/total volume ratio (BV/TV). Dashed red lines correspond to the empty defect threshold (i.e., group 3). (*n* = 9, Mann–Whitney test).

It was reported that an increase in the production of pro-inflammatory cytokines leads to faster degradation of the material along with an acceleration in bone formation [29]. Based on the 3Rs principles as well as practical issues, rat parietal (i.e., calvarial) bone defect model was used to demonstrate the ability of the DCPD-CHI-HA functionalized Bio-Gide^®^ membrane to induce bone regeneration. For that, three groups were created: group 1 (9 rats) received the DCPD-CHI-HA functionalized Bio-Gide^®^ membrane, group 2 (9 rats) received the raw Bio-Gide^®^ membrane and group 3 (3 rats) had an empty defect as a negative control. The rats were constantly monitored after the surgery until recovery. Gross observation of animals post-surgery in all groups did not show any complications. The DCPD-CHI-HA membranes exhibited a good tissue compatibility and no adverse events such as a foreign body reaction nor infection were observed in group 1 throughout the study period of eight weeks (Figure 5A). Microtomography (μ–CT) analysis was carried out for the structural and quantitative assessments of the de novo bone. No hard-tissue coverage of group 3 (i.e., control defect) was observed even after eight weeks of post-surgery (Figure S3—SI). μ–CT coronal views showed almost complete closure of the defect in group 1 in comparison with group 2 (Figure 5B), while μ–CT trans-axial views revealed an increase in bone volume in group 1 and a limited bone formation in contact with dura in the group 2 (Figure 5C). To confirm these results, quantitative analyzes of bone volume and bone volume/total volume ratio were performed. The results revealed that the DCPD-CHI-HA group significantly out-performed the raw membrane group in terms of bone volume (1.58 fold increase, *p* < 0.004, Figure 5D,E). Taken together, these results demonstrated that the DCPD-CHI-HA is a biocompatible and osteoinductive material with a successful stimulating bridging of the bone defect as well as an increase in the mineralized bone volume.

**Figure 6:**
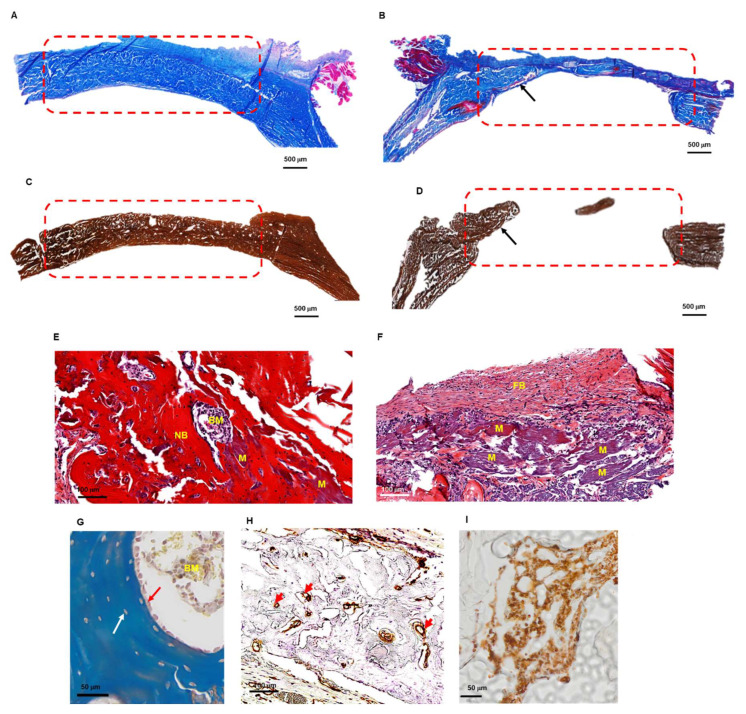
Histological analysis of 8-weeks de novo bone. (**A**–**D**): Resin-embedded sections of the de novo calcified parietal bone in the presence of DCPD-CHI-HA (group 1, (**A**,**C**)) and the raw membranes (group 2, (**B**,**D**)), stained with Masson’s trichrome (**A,B**) and Von Kossa (**C**,**D**). Dashed rectangles, highlighting the region of interest, showed the increase in bone volume in group 1 (Scale bars = 500 μm). Black arrows indicate the in-growth pattern from the peripheral region in group 2. (**E**–**G**): Paraffin-embedded sections of the de novo decalcified parietal bone in the presence of DCPD-CHI-HA (group 1, (**E**,**G**)) and the raw membranes (group 2, (**F**)), stained with hematoxyline-eosin-safran (**E**,**F**) and Masson’s trichrome (**G**), indicating the presence of osteoblasts (red arrow) and osteocyte lacuna (white arrow); BM = bone marrow, NB = new bone, M = remanent membrane, FB= fibrotic tissue red and white arrows; scale bars = 100 μm and 50 μm. (**H**,**I**): CD31 and CD68 immunohistochemical staining of paraffin-embedded sections of the de novo decalcified parietal bone in the presence of DCPD-CHI-HA, indicating respectively the presence of blood vessels (red arrows; scale bar = 100 μm) and the presence of macrophages in bone marrow (Scale bar = 50 μm).

Masson’s trichrome (MT) and von Kossa staining were used to further evaluate de novo bone patterns on resin embedded-calcified parietal bones (Figure 6A,B). The thickness bridging in the bone defect site confirmed the superiority of the DCPD-CHI-HA compared to the raw Bio-Gide^®^ membrane. The bone tissue in-growth pattern from the defect edges to its center was observed, a signature of osteoconductive and osteoinductive features of DCPD-CHI-HA [14]. We noticed that most of the defect regions in group 1 seem filled with a mature-*like* bone tissue, while group 2 displayed a bone tissue in-growth pattern from the peripheral region. Only a small amount of thin new bone and loose connective tissue in the central part was observed. Furthermore, in contrast to the previously described μ–CT results, the histological examination did not reveal the de novo bone on the dura side. The Bio-Gide^®^ membrane is mainly constituted of type I collagen that has the potential to be mineralized [32]. Therefore, to evaluate the absence of heterotopic mineralization and the resorption of the membrane, sections of the paraffin-embedded-decalcified specimens were stained with hematoxylin-eosin-safran (HES). Observations did not reveal abnormal infiltration of inflammatory cells in the healed defects and around the membrane remnants. HES staining confirmed the de novo bone formation in group 1, which seems to originate from the dura side and extended toward the periosteal side of the defect (Figure 6C,D). Furthermore, in the presence of DCPD-CHI-HA the Bio-Gide^®^ membranes were almost completely degraded while substantial fragments of the raw membranes were still visible in group 2 (Figure 6E,F). In group 1, the de novo bone was marked by the presence of osteoblasts, osteocyte lacunae, bone marrow and blood vessels confirmed by CD31 positive cells (i.e., endothelial cell marker) (Figure 6E–H). CD68 positive cells (i.e., macrophage marker) were mainly localized in bone marrow (Figure 6I).

**Figure 7:**
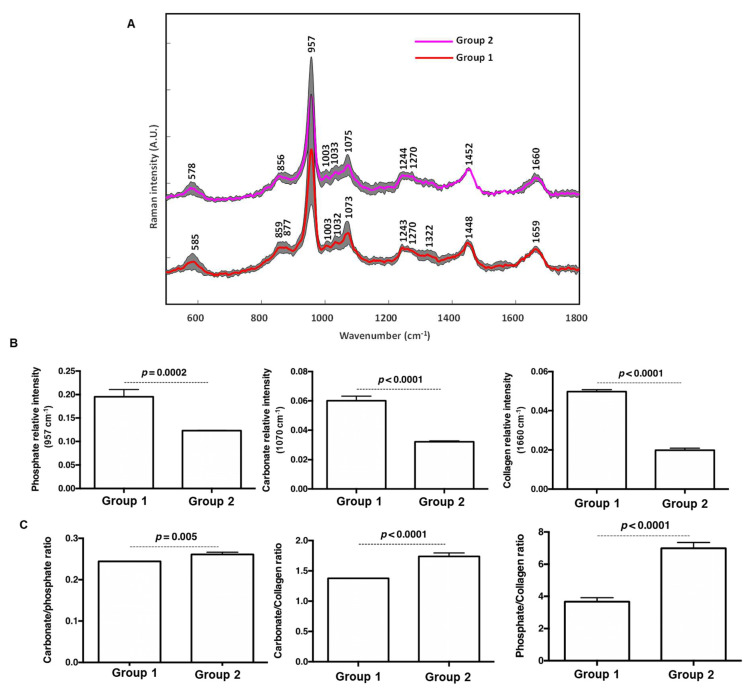
Mineral chemistry of 8-weeks de novobone. (**A**): Raman spectra of parietal bone formed in the presence of DCPD-CHI-HA (group 1) and the raw membranes (group 2). (**B**): Relative band intensity of the main constituent of bone (phosphate, carbonate and collagen), indicating higher intensities in group 1 in comparison with group 2. (**C**): Ratios of Carbonate/Phosphate, Carbonate/Collagen and Phosphate/Collagen, indicating higher de novo bone in group 1 (*n* = 9, Mann–Whitney test).

We corroborated our exciting previous observations by bone mineral chemistry analysis with Raman spectroscopy. Raman spectroscopy analysis provides several advantages such as real-time analysis of thick, fresh and hydrated specimens. The resulting spectra (Figure 7A) performed on the freshly explanted specimen after eight weeks of implantation revealed the presence of bone-specific bands: 586 cm^−1^ (ʋ4 PO_4_^3−^), 957 cm^−1^ (ʋ1 PO_4_^3−^) and 1070 cm^−1^ (ʋ1 CO_3_^2−^), responsible for vibrations of carbonated calcium phosphate in an apatitic lattice. The peaks at 1244 cm^−1^ and 1270 cm^−1^ (amide III), 1452 cm^−1^ (CH2 bending/deformation) and 1660 cm^−1^ (amide I) are related to collagen matrix vibrations. Relative intensities of 957 cm^−1^ and 1070 cm^−1^ bands were used to investigate the changes in mineral components between group 1 and group 2 (Figure 7B). Raman intensity of 1660 cm^−1^ band was used to evaluate the relative contribution of the organic matrix between these groups. Relative band intensities of phosphate and carbonate were higher in group 1 in comparison with group 2. Type A and B carbonate substitutions are located at 1103 and 1070 cm^−1^ respectively [21]. The presence of the band at 1070 cm^−1^ and the absence of the band around 1103 cm^−1^ in Raman spectra indicates a major role of B-type carbonate apatite. Although in group 2 the formation of fibrotic connective tissue within the defect was observed (Figure 6F), the relative intensity collagen band at 1660 cm^−1^ was higher in group 1. The mineral to matrix ratio, used to estimate the degree of bone mineralization, is a compositional Raman parameter linked to bone mechanical strength. Carbonate-to-phosphate ratio was lower in group 1 in comparison to group 2 (Figure 7C); this could be attributed to incomplete bone healing/regeneration process in the presence of the raw membrane [33]. Furthermore, mineral-to-collagen ratios (i.e., carbonate-to-collagen and phosphate-to-collagen) were higher in the de novo bone in the presence of DCPD-CHI-HA versus raw Bio-Gide^®^ membrane. To sum up, an increase in the mineral/matrix ratios in the presence of DCPD-CHI-HA could result in a more mature-*like* bone with an increase in the bone mechanical strength [34].

**Figure 8:**
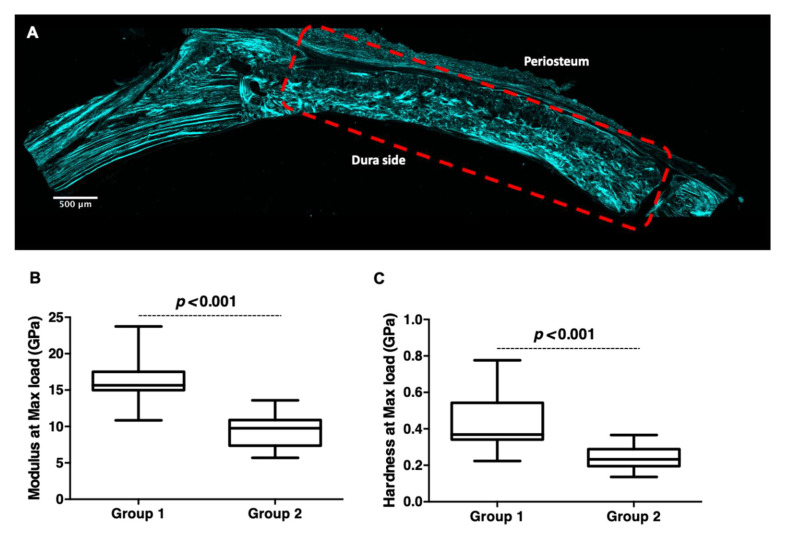
Mechanical behavior of 8-weeks de novo bone. (**A**): Confocal microscopy-Second Harmonic Generation, indicating the collagen organization. Dashed rectangles indicate the region of interest (scale bar = 500 μm). (**B**,**C**): Modulus and Hardness at max load, respectively, measured by nanoindentation. (*n* = 4, Mann–Whitney test).

During bone healing, the first produced bone is characterized by a disorganized arrangement of collagen fibers and thus weak mechanical properties, called osteoid [35]. Following the bone remodeling process, the newly formed bone is replaced by regular, parallel aligned collagen fibers that form sheet-*like* structures called lamellar bone. In this study, laser scanning confocal microscopy and second harmonic generation (SHG) imaging showed a few regions with lamellar-*like* collagen assembly in group 1 (Figure 8A); this organization was predominant in the dura side. In contrary, the SHG signal was not detected in the group 2 defect (Figure S4—SI).

As in the presence of DCPD-CHI-HA, the formation of a more mature bone (i.e., an increase in bone volume with the formation of a lamellar-*like* collagen assembly) was enhanced, we hypothesized that the de novo bone in the presence of the DCPD-CHI-HA membrane would be stiffer. To assess this, we evaluated at nanoscale the mechanical performance of the de novo bone. The nanoindentation in the center of the sample revealed that the modulus and the hardness at the max load were significantly higher for the group 1 compared to the group 2 (*p* < 0.001 for both, Figure 8B,C), reflecting therefore an increase in the stiffness in the presence of DCPD-CHI-HA.

## 7. Conclusions

In summary, the Bio-Gide^®^ membrane was functionalized with bioactive dicalcium phosphate dihydrate, chitosan and hyaluronic acid able to release calcium and phosphorus ions in biological fluids. In vitro inflammatory studies revealed an increase in inflammatory mediator release by macrophages in the presence of the functionalized membrane in comparison with the raw membrane. In vivo studies demonstrated that the DCPD-CHI-HA membrane out-performed the raw membrane with regards to de novo bone in a critical-sized defect in rat calvaria model. The in-depth analysis after eight weeks of implantation showed that de novo bone displayed a physiological matrix composition with collagen and hydroxyapatite mineral and the quasi absence of the implanted membrane; this study showed the potential of dicalcium phosphate dihydrate, chitosan and hyaluronic acid as a functional membrane for GBR application and demonstrated the utility of adopting a thorough multi-scale materials-based characterization approach when investigating bone substitute performances.

## Data Availability

Not applicable.

## Supplementary Materials

The following supporting information can be downloaded at: https://www.mdpi.com/article/10.3390/cells11182865/s1, Figure S1: Raman spectra map showing the spatial distribution of the mineral-to-organic ratio (I988/I1670) revealed het-erogeneity in the distribution of mineral products on the Bio-Gide^®^ collagen membrane; Figure S2: FEG-SEM views of CD14+ monocytes in the contact with glass and in the presence of LPS stimulus (Scale bar = 1 μm), highlighting the morphology of the activated monocyte; Figure S3: The parietal bone recovery of the empty bone defect (group 3). Macroscopical examination (A), micro-CT coronal (B) and transaxial (C) views showing the absence of the de novo bone (scale bars = 1 mm).; Figure S4: Confocal microscopy-Second Harmonic Generation, indicating the absence of the collagen organization within the recovered bone defect side (group 2). Dashed rectangles indicate the region of interest (scale bar = 500 μm).
