# Supplementary material for: Hybrid Mineral/Organic Material Induces Bone Bridging and Bone Volume Augmentation in Rat Calvarial Critical Size Defects
Source: Cells. 2022 Sep 14;11(18):2865. doi: 10.3390/cells11182865 (PMC9497222; doi:10.3390/cells11182865)
Supplement: Supplementary file 1 [file cells-11-02865-s001.zip › cells-1828553-supplementary.pdf]

cells

Hybrid Mineral/Organic Material Induces Bone Bridging and Bone Volume Augmentation in rat Calvarial Critical Size Defects

Marie Dubus[1,2], Loïc Scomazzon[1], Charlotte Ledouble[1,2,3], Julien Braux[1,2,3], Abdelilah Beljebbar[4,5], Laurence Van Gulick[4], Adrien Baldit[6], Caroline Gorin[7,8], Halima Alem[9], Nicole Bouland[10], Marissa Britton[11], Jessica Schiavi[11], Ted Vaughan[11], Cedric Mauprivez[1,2,3] and Halima Kerdjoudj[1,2]

1. Université de Reims Champagne Ardenne, EA 4691, Biomatériaux et Inflammation en Site Osseux (BIOS), Reims, France.
2. Université de Reims Champagne Ardenne, UFR d'Odontologie, Reims, France.
3. Centre Hospitalier Universitaire de Reims, Pôle Médecine Bucco-Dentaire, Hôpital Maison Blanche, France.
4. Université de Reims Champagne Ardenne, EA 7506, BioSpecT, Reims, France.
5. Université de Reims Champagne Ardenne, UFR de Pharmacie, Reims, France.
6. Université de Lorraine, ENIM, CNRS, LEM3, Metz, France.
7. Université Paris Cité, URP2496 Pathologies, Imagerie et Biothérapies Orofaciales et Plateforme Imagerie du Vivant, UFR Odontologie, Paris, France.
8. AP-HP, Services Médecines bucco-dentaire (GH Paris Sud-Sorbonne Université), France.
9. Université de Lorraine, CNRS, IJL, Nancy, France.
10. Université de Reims Champagne Ardenne, Service d'Anatomo-Pathologie, Reims, France.
11. Biomechanics Research Centre (BioMEC), Biomedical Engineering, School of Engineering, College of Science and Engineering, National University of Ireland, Galway, Ireland.

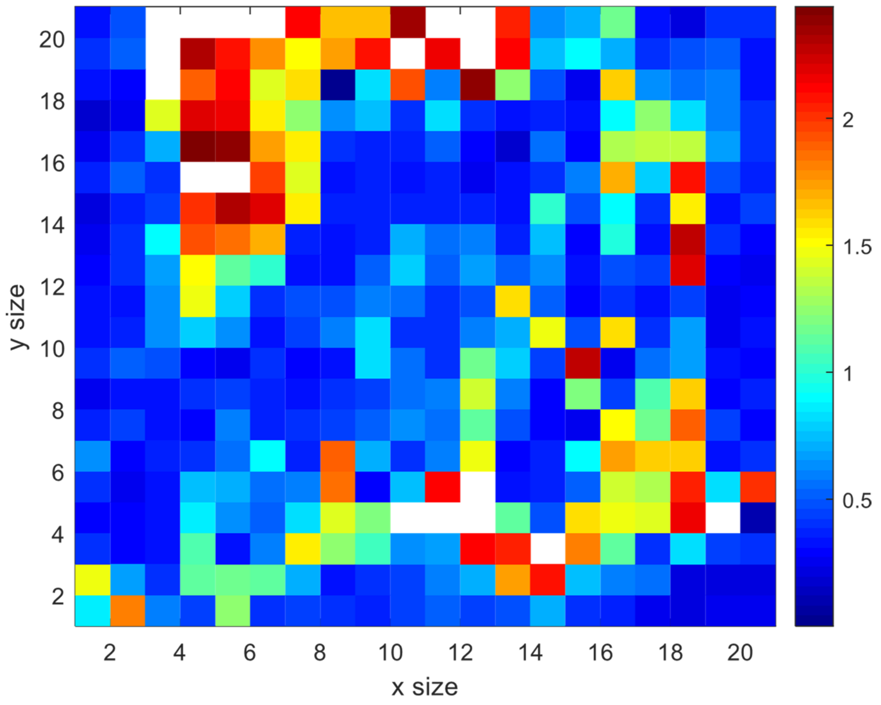

Figure S1: Raman spectra map showing the spatial distribution of the mineral-to-organic ratio (I_{988}/I_{1670}) revealed heterogeneity in the distribution of mineral products on the Bio-Gide® collagen membrane.

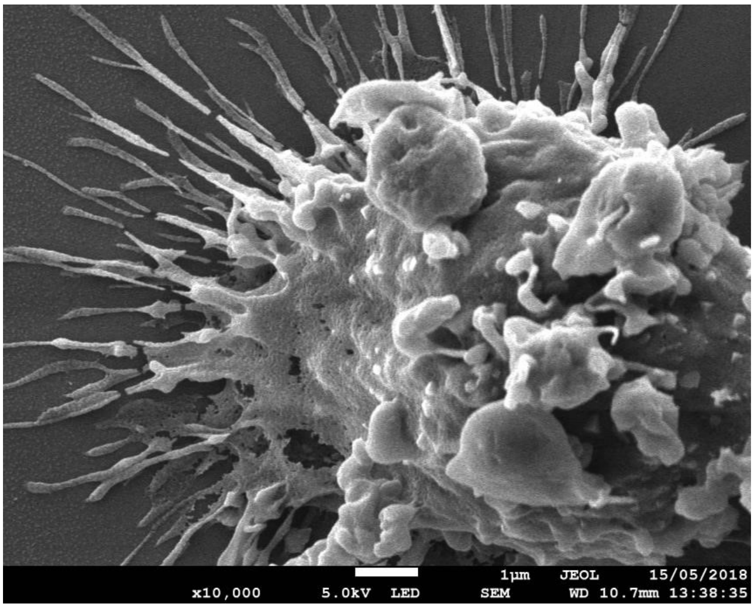

Figure S2: FEG-SEM views of CD14⁺ monocytes in the contact with glass and in the presence of LPS stimulus (Scale bar = 1 m), highlighting the morphology of the activated monocyte.

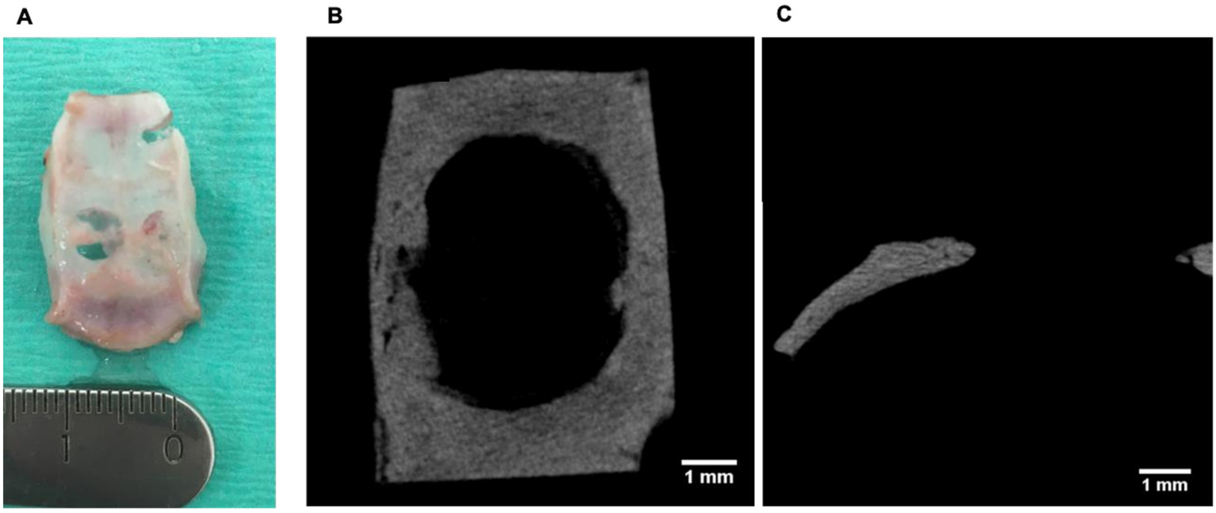

Figure S3: The parietal bone recovery of the empty bone defect (group 3). Macroscopical examination (**A**), micro-CT coronal (**B**) and transaxial (**C**) views showing the absence of the *de novo* bone (scale bars = 1 mm).

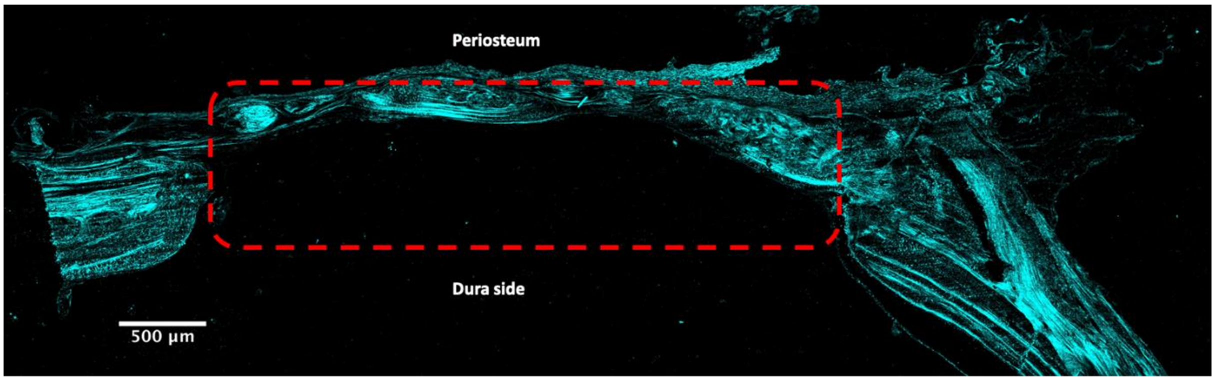

Figure S4: Confocal microscopy-Second Harmonic Generation, indicating the absence of the collagen organization within the recovered bone defect side (group 2). Dashed rectangles indicate the region of interest (scale bar = 500 µm).
